# Potassium binders in clinical practice: understanding potassium binder use in contemporary Swedish healthcare—the DEMONSTRATE database

**DOI:** 10.1186/s12882-025-04146-8

**Published:** 2025-04-28

**Authors:** Hans Furuland, Anders Olof Larsson, Per Bjellerup, Milica Uhde, Thomas Cars, Matilda Almstedt, Maria K. Svensson

**Affiliations:** 1 Department of Medical Sciences, Renal Medicine, Uppsala University Hospital, Akademiska Sjukhuset, Entrance 40, Floor 5, Uppsala, SE 751 85 Sweden; 2 Department of Medical Sciences, Clinical Chemistry, Uppsala University Hospital, Uppsala, Sweden; 3 Department of Clinical Research Center, Uppsala University, Västerås, Uppsala, Sweden; 4 Department of Laboratory Medicine, Central Hospital Västmanland, Västerås, Sweden; 5 Vifor Pharma Nordiska AB, Solna, Sweden; 6 Sence Research AB, Uppsala, Sweden; 7 Uppsala Clinical Research Centre, Uppsala, Sweden

**Keywords:** Hyperkalemia, Potassium binders, RAASi treatment, Real-world data, Sweden

## Abstract

**Background:**

Potassium binders mitigate hyperkalemia, allowing patients to maintain their renin-angiotensin-aldosterone-system inhibitor (RAASi) treatment. This study characterized patients treated with first- or second-generation potassium binders, usage patterns and their effectiveness in reducing potassium levels, and changes in RAASi treatment in a Swedish population-based study.

**Methods:**

A *National Cohort* included patients who had record of a treatment episode with a first-generation or second-generation potassium binder between 2018 and 2022. A *Mid-Sweden Cohort* included patients from the *National Cohort* who also had a record of a potassium measurement within the 60 days prior to beginning potassium binder treatment. Comorbidities, prior medication use, persistence with potassium binder treatment, subsequent changes in potassium levels and RAASi treatment were evaluated. Persistence was analyzed using the Kaplan-Meier estimator and changes in potassium levels were assessed using linear mixed-effects models.

**Results:**

23,892 treatment episodes involving 14,235 patients (mean age 70 years, 33% women) were followed in the *National Cohort*, and 4860 episodes involving 3179 patients (mean age 72 years, 34% women) in the *Mid-Sweden Cohort*. Patients treated with second-generation potassium binders had more comorbidities and higher median persistence with treatment compared to those on first-generation potassium binders, 112.5 (95% CI:112.5-117.5) vs. 87.5 (95% CI: 87.5–87.5) days in the *National Cohort*; 165.5 (95% CI: 121.0-198.0) vs. 97.6 (95% CI: 87.5–110.0) days in the *Mid-Sweden Cohort*. Both first- and second-generation potassium binders reduced potassium levels from baseline by day 15, 5.7 [95% CI: 4.5–6.8] mmol/L to 4.7 [95% CI: 3.6–5.9] mmol/L and 5.5 (95% CI: 4.3–6.7) mmol/L to 4.9 (95% CI: 3.8–6.1) mmol/L, respectively. Dose reduction or discontinuation of renin-angiotensin system inhibitors (RASi) or mineralocorticoid receptor antagonists (MRAs) was found in 31.4% and 47.7%, respectively, within 120 days of initiating therapy.

**Conclusion:**

Both potassium binders effectively reduced potassium levels, but frequent discontinuation or dose reduction of RAASi therapy were still observed during this period. The adjustments of RAASi therapy, despite the achievement of normokalemia within 15 days, may be premature and warrants careful reconsideration to ensure optimal patient outcomes.

**Supplementary Information:**

The online version contains supplementary material available at 10.1186/s12882-025-04146-8.

## Introduction

Renin-angiotensin aldosterone system inhibitors (RAASi) are widely prescribed to patients with hypertension, heart failure, diabetes, and chronic kidney disease (CKD). While the use of RAASi reduces the risk of major adverse cardiovascular events and all-cause death in these patients [1], it may increase the risk of hyperkalemia [2, 3], a condition where serum potassium levels are elevated, potentiating cardiac dysrhythmias and sudden cardiac death [4]. The risk of hyperkalemia is particularly high in patients with CKD [2].

Traditionally, hyperkalemia in patients using RAASi has often been addressed by discontinuing RAASi treatment or by reducing the dose [3]. Yet, such treatment modifications are potentially associated with increased cardiovascular and mortality risks [4, 5]. First- and second-generation potassium binders have demonstrated effectiveness in reducing serum potassium levels in clinical studies [6–12], and may mitigate the risk of hyperkalemia and allow these patients to maintain their RAASi treatment. The second-generation potassium binders, introduced in Sweden between 2018 and 2019, were mainly developed due to concerns regarding the gastrointestinal side-effects of first-generation potassium binders. There is a need to evaluate the use of potassium binders in contemporary routine care and its influence on RAASi treatment.

This study used nationwide and regional data sources to characterize a contemporary patient population treated with potassium binders and assessed their patterns of use of the medication. Additionally, this study aimed to evaluate the effectiveness of first- and second-generation potassium binders in reducing serum potassium levels and to assess changes in RAASi use following the initiation of potassium binder treatment.

## Methods

### Data sources

This study was conducted in Sweden, where residents have access to a comprehensive nationwide public healthcare system with minimal co-payments for healthcare visits, hospitalizations, and medications [13]. Each resident’s interactions with the healthcare system, including the filling of prescriptions, are recorded using a unique personal identification number, providing nearly complete population-wide medical history.

Data were extracted from multiple sources: the National Patient Register, the Swedish Prescribed Drug Register, the Cause of Death Register, and clinical chemistry departments in six mid-Sweden counties (Uppsala, Gävleborg, Västmanland, Sörmland, Värmland, and Örebro), collectively representing approximately 18% of Sweden’s population (1.86 million residents). The data from each source were linked for each patient using the personal identification number, further pseudonymized, and compiled to form the DEMONSTRATE database. To our knowledge, this is the first study to integrate national health records with regional clinical chemistry data from such an extensive network of departments.

The study was conducted in accordance with the Declaration of Helsinki and was approved by the Swedish Ethical Review Authority (approval number 2022-01051-01 and 2022-06306-02). Given the nature of the study, informed consent was not required.

### Patients

A *National Cohort* was established, comprised of patients across Sweden who had record of treatment episodes with potassium binders between January 1st, 2018, and December 31st, 2022. Records of treatment with first-generation (sodium polystyrene sulfonate) and second-generation (patiromer, sodium zirconium cyclosilicate) potassium binders were included (Supplemental Methods 1). While sodium polystyrene sulfonate has been prescribed in Sweden for decades, second-generation potassium binders have only been available since late 2018 [14].

Additionally, a *Mid-Sweden Cohort* was formed, consisting of a subset of patients from the *National Cohort* who also had potassium measurements recorded at any of the six clinical chemistry departments within 60 days prior to, or on the day of, the start of potassium binder treatment. The start of the treatment episode was designated as the index date for follow-up in both cohorts. Patients could have multiple treatment episodes and could therefore be followed more than once (Supplemental Methods 2).

### Patient characteristics

Diagnoses recorded in any position in primary care, specialized outpatient care, and/or inpatient care prior to and on the index date were used to identify ongoing and prevalent comorbidities (Supplemental Methods 3). For the *Mid-Sweden Cohort*, the highest serum potassium concentration measured within the 60 days prior to, or on, the index date was used to assess baseline potassium levels. Kidney function was estimated using the most recent estimated glomerular filtration rate (eGFR) value prior to the index date, calculated with the revised Lund-Malmö equation. Medication use was determined based on prescriptions filled during the 120 days prior to or on the index date (Supplemental Methods 4).

### Persistence with potassium binder treatment (treatment episodes)

The duration of the treatment episodes with first- or second-generation potassium binders was assessed in both the *National Cohort* and the *Mid-Sweden Cohort*. The duration of medication supply was calculated by dividing the total quantity dispensed within the episode by the prescribed daily dose, which was determined by semi-manually analyzing the free-text prescribed dose. If the prescribed dose could not be interpreted for a specific potassium binder dispensation, the mean dose from all interpretable prescriptions of that binder was imputed. To account for variation in adherence, a 25% grace period was added to each pharmacy dispensation of potassium binder [15]. The proportion of patients who remained on potassium binder treatment was assessed daily for up to three years from the index date and presented using Kaplan-Meier plots. Follow-up ended at the end of the study (31 December 2022), death, a treatment switch between first- and second-generation potassium binders, or with treatment discontinuation (outcome).

### Changes in potassium levels

A mixed-effects model was developed using data from the *Mid-Sweden Cohort* to assess changes in potassium levels following treatment with first- and second-generation potassium binders. The model included treatment type (first- or second-generation potassium binders), time (modeled as a restricted cubic spline with four knots), their interaction, and the following baseline characteristics: age, sex, heart failure, hypertension, chronic kidney disease, diabetes mellitus, baseline potassium levels, and use of renin-angiotensin system inhibitors (RASi) and/or mineralocorticoid receptor antagonists (MRAs).

Two separate models were fitted: one for pre-treatment potassium values and one for post-treatment values. The mixed-effects model was further used to predict potassium levels in the *Mid-Sweden Cohort* at baseline and at 15-, 30-, 45-, and 60-days post-treatment initiation. Results are presented with all measured potassium values plotted as individual data points and predicted estimates for the median patient (male, age 69, with hypertension, CKD, and RAASi treatment) at specified time points (0, 15, 30, 45, and 60 days), including 95% confidence intervals. This analysis was restricted to patients who a minimum of 60 days of follow up. In a sensitivity analysis, we conducted an as-treated analysis that included only potassium values measured while patients had an active supply of potassium binder treatment at the time of their potassium measurement (Supplemental Fig. 1).

### Changes in renin-angiotensin aldosterone system inhibitor use

Changes in RAASi use following potassium binder initiation were assessed in the *National Cohort*, which included the *Mid-Sweden Cohort*. Patients who filled a prescription for an angiotensin-converting enzyme inhibitor (ACEi), angiotensin receptor blocker (ARB), or MRA within 120 days prior to or on the index date were included. The analysis was restricted to potassium binder treatment episodes with at least 120 days of follow-up.

The proportion of patients who filled a RAASi prescription was calculated for two consecutive 120-day periods post-index (days 1-120 and 121–240). Changes in RAASi use were categorized as discontinuation, dose reduction, dose increase, or continuation of the current dose (Supplemental Methods 5).

## Results

### Patient characteristics

The *National Cohort* included 14,235 patients (70 years, 33% women), from which a total of 23,892 potassium binder treatment episodes could be followed. The *Mid-Sweden Cohort* (a subset of the *National Cohort)* consisted of 3179 patients (72 years, 34% women) with 4860 potassium binder treatment episodes. Clinical characteristics at the index date were similar between the two cohorts (Table 1). About one-third of patients had heart failure, nearly 90% had hypertension, almost half had diabetes, and more than 80% had CKD.

**Table 1:** Clinical characteristics of patients at the beginning of each potassium binder treatment episode

		National cohort	Mid-Sweden cohort
**N, potassium binder treatment episodes**		23,892	4860
**N, patients**		14,235	3179
**Age, years**	Mean (SD)	70.2 (15.0)	71.6 (14.6)
	Median (IQR)	73.0 (62.0–81.0)	74.0 (64.0–82.0)
**Women**,** N (%)**		7913 (33.1%)	1653 (34.0%)
**Laboratory measurements**			
Baseline potassium level (mmol/L)	Mean (SD)	N/A	5.6 (0.7)
eGFR, ml/min/1.73m^2^	Mean (SD)	N/A	23.6 (17.1)
	Median (IQR)	N/A	19.0 (10.3–31.1)
**Comorbidities**,** N (%)**			
Heart failure		8325 (34.8%)	1876 (38.6%)
Hypertension		20,794 (87.0%)	4298 (88.4%)
Diabetes mellitus		10,867 (45.5%)	2272 (46.7%)
Type 1 diabetes mellitus		5006 (21.0%)	1081 (22.2%)
Type 2 diabetes mellitus		10,306 (43.1%)	2179 (44.8%)
Chronic kidney disease (based on diagnosis and procedure codes)		19,976 (83.6%)	3950 (81.3%)
eGFR < 60 ml/min/1.73m^2^		N/A	4632 (95.3%)
eGFR < 15 ml/min/1.73m^2^		N/A	1901 (39.1%)
**Dialysis**,** N (%)**			
Hemodialysis		6295 (26.3%)	1155 (23.8%)
Peritoneal dialysis		914 (3.8%)	165 (3.4%)
**Medication use**,** N (%)**			
RASi (ACEi, ARB)		14,836 (62.1%)	3197 (65.8%)
RAASi (ACEi, ARB, MRA)		15,396 (64.4%)	3368 (69.3%)
ARNi		485 (2.0%)	129 (2.7%)
MRA		2996 (12.5%)	888 (18.3%)
Antihypertensive medications		21,335 (89.3%)	4435 (91.3%)
Diuretics		11,770 (49.3%)	2400 (49.4%)
Beta Blockers		15,549 (65.1%)	3350 (68.9%)
SGLT2i		666 (2.8%)	128 (2.6%)

eGFR, estimated glomerular filtration rate; RASi, renin-angiotensin system inhibitors; RAASi, renin-angiotensin-aldosterone system inhibitors; ACEi, angiotensin-converting enzyme inhibitors; ARB, angiotensin receptor blockers; ARNi, angiotensin receptor-neprilysin inhibitors; angiotensin receptor-neprilysin inhibitors; MRA, mineralocorticoid receptor antagonists; SGLT2i, sodium-glucose cotransporter-2 inhibitors

Approximately 30% of patients had a history of dialysis, either currently undergoing treatment with hemodialysis or peritoneal dialysis, or having previously received dialysis.

Nearly 90% were treated with antihypertensive medications, two-thirds with RAASi, two-thirds with beta-blockers, and almost half with diuretics. Less than 3% were treated with angiotensin receptor-neprilysin inhibitors (ARNi) or sodium-glucose cotransporter-2 inhibitors (SGLT2i).

**Fig. 1:**
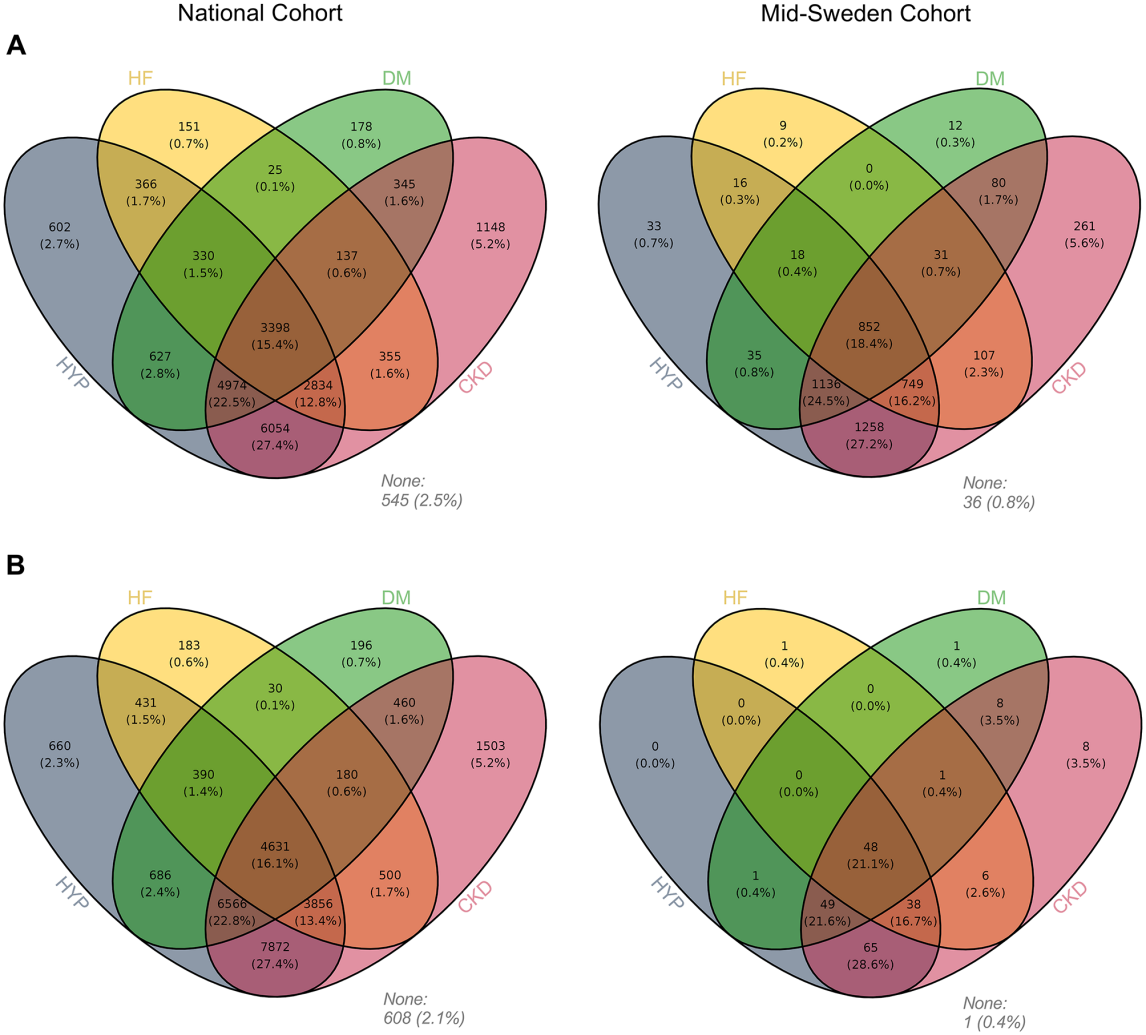
Overlapping comorbidities for patients who were treated with (**A**) first- or (**B**) second-generation potassium binders. The analysis was performed separately for patients included in the *National Cohort* and patients included in the *Mid-Sweden Cohort*. Four comorbidities, including heart failure (HF), diabetes mellitus (DM), hypertension (HYP), and chronic kidney disease (CKD), were included in the analysis

Differences were observed in patient characteristics between those treated with first- and second-generation potassium binders within both cohorts (Table S1). Patients who received second-generation binders were, on average, younger and had higher proportions of heart failure and CKD. Despite these differences, in an analysis of overlapping comorbidities (Fig. 1), the proportions of patients in each cohort who had a combination of heart failure, diabetes mellitus, hypertension, and CKD were similar between patients treated with a first and those treated with a second-generation potassium binder. A larger proportion of the patients treated with a second-generation potassium binder were on dialysis.

There were also distinct differences in characteristics between patients treated with RAAS inhibitors at index and those who were not (Table S2). Patients on RAASi generally had higher rates of heart failure, hypertension, type 1 and type 2 diabetes, but lower rates of CKD and dialysis treatment. Those who used RAASi were also more frequently treated with diuretics, beta-blockers, and SGLT2i.

There were similar differences in characteristics between patients treated with an MRA at index and those who were not (Table S3). Patients who used an MRA more often had heart failure and type 2 diabetes, but less of type 1 diabetes, CKD, and dialysis treatment. Those who used an MRA were also more frequently treated with ARNi, diuretics, beta-blockers, and SGLT2i.

### Persistence with potassium binder treatment

**Fig. 2:**
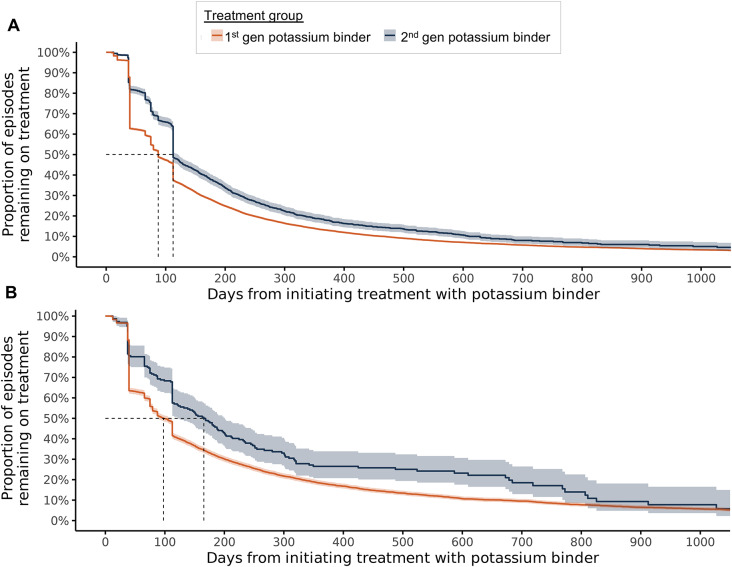
Persistence of treatment with potassium binders for patients in (**A**) *National* and (**B**) *Mid-Sweden Cohort*

In both the *National Cohort* and the *Mid-Sweden Cohort*, persistence (i.e., the proportion of patients remaining on medication) was higher among those treated with second than those treated with first-generation binders (Fig. 2). In the *National Cohort*, the median treatment duration was 87.5 days (95% CI: 87.5–87.5) for first-generation binders versus 112.5 days (95% CI: 112.5-117.5) for second-generation binders. In the *Mid-Sweden Cohort*, the median duration was 97.6 days (95% CI: 87.5–110.0) for first-generation binders compared to 165.5 days (95% CI: 121.0-198.0) for second-generation binders.

### Changes in potassium levels

**Fig. 3:**
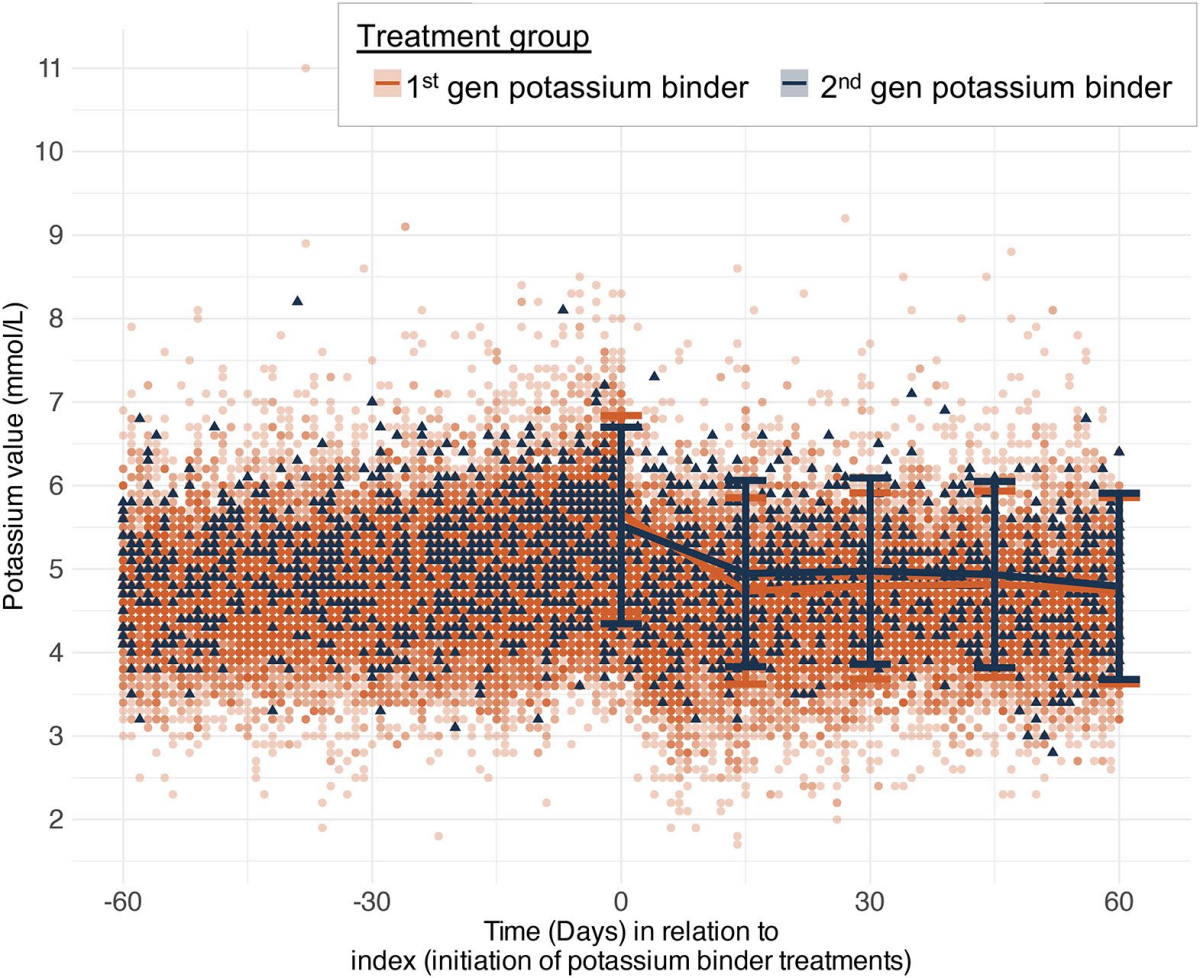
Changes in potassium in Mid-Sweden Cohort during 60 days after initiating potassium binder treatment. The scatter plot shows individual potassium measurements over time in patients treated with first- or second-generation potassium binders. The solid lines represent predicted potassium levels derived from a mixed-effects model adjusted for age, sex, heart failure, hypertension, chronic kidney disease, diabetes, use of RASi and MRA, and baseline potassium levels. Prediction mean with error bars reflecting 95% confidence intervals are presented for the median patient at index and 15-, 30-, 45-, and 60-days post-index)

Figure 3 illustrates the mixed-effects model showing both the measured and predicted potassium values for patients in the *Mid-Sweden Cohort* treated with first- and second-generation potassium binders. For first-generation binders, baseline potassium levels decreased from 5.7 mmol/L (95% CI: 4.5–6.8) to 4.7 mmol/L (95% CI: 3.6–5.9) by the 15th day following treatment initiation, while for second-generation binders, potassium levels decreased from 5.5 mmol/L (95% CI: 4.3–6.7) to 4.9 mmol/L (95% CI: 3.8–6.1) within the same time period. Following this initial reduction, potassium levels remained stable throughout the remaining 45 days of follow-up. A similar pattern was observed in the as-treated analysis, with potassium levels remaining stable over the follow-up period.

### Changes in renin-angiotensin aldosterone system inhibitor use

**Fig. 4:**
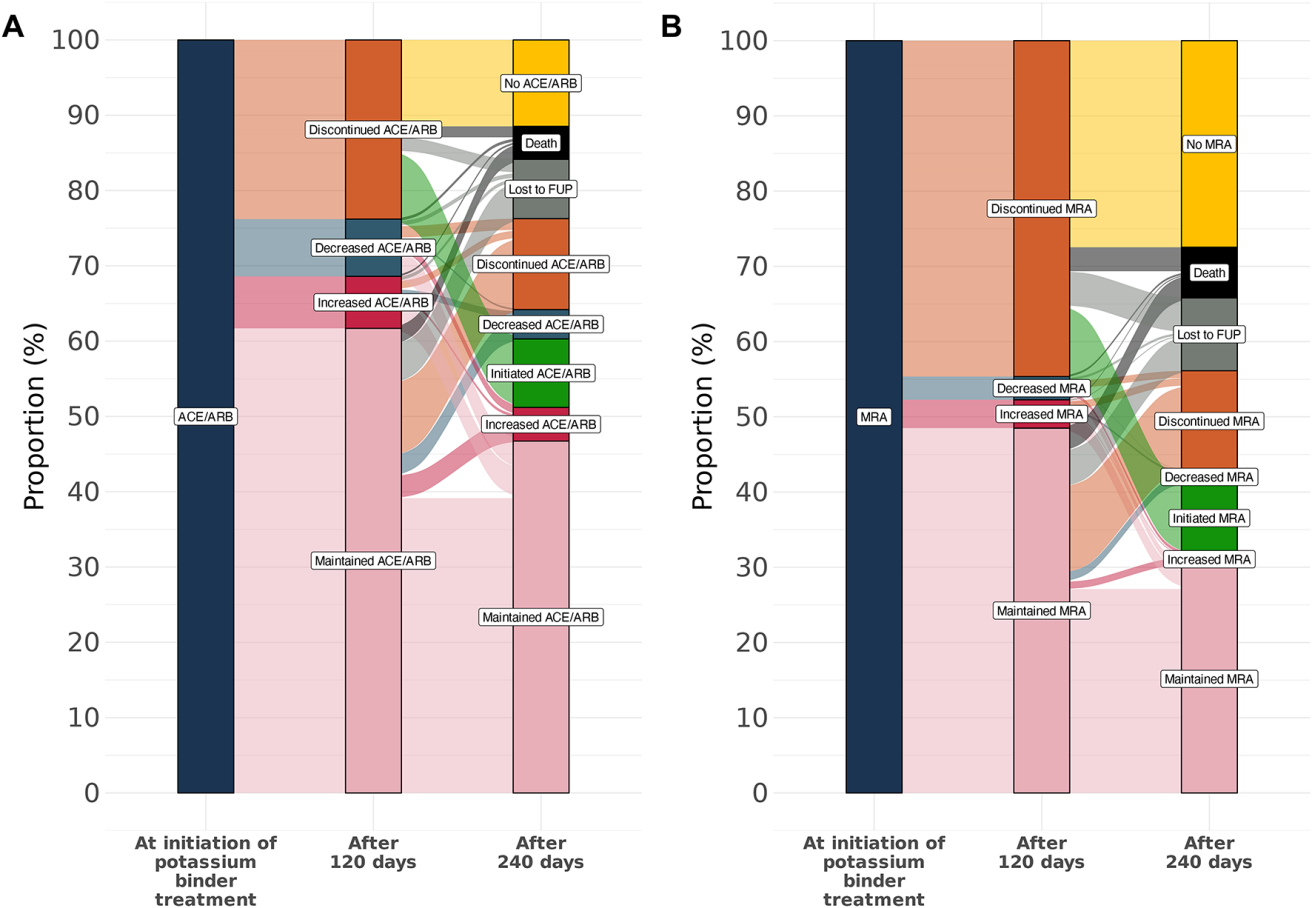
Changes in RAASi treatment in National Cohort during 240 days after initiating potassium binder treatment. ACE, angiotensin-converting enzyme inhibitors; ARB, angiotensin receptor blockers; FUP, follow-up; MRA, mineralocorticoid receptor antagonists. Changes in RAASi treatment in *National Cohort* during 240 days after initiating potassium binder treatment

Among the 23,892 potassium binder treatment episodes in the *National Cohort*, 11,929 were treated with an ACEi or ARB at index, with at least 120 days of follow-up available. In about one-third (31.4%) of these episodes, ACEi or ARB treatment was either discontinued or the dose was reduced within the first 120 days (Fig. 4A). The dose was increased in 6.9% of episodes, while ACEi or ARB therapy was maintained in 62.0% of episodes.

During the subsequent 120-day period, ACEi or ARB treatment was further discontinued or reduced in an additional 16.0% of episodes. Among those who discontinued ACEi or ARB treatment in the initial 120 days, 37.8% re-initiated therapy in the following 120 days.

Similarly, there were 2229 episodes where patients were treated with a MRA at index with at least 120 days of follow-up. In nearly half (47.7%) of these episodes, MRA treatment was discontinued, or the dose reduced within the first 120 days (Fig. 4B). In the subsequent 120 days, MRA treatment was further discontinued, or the dose reduced in 14.9% of episodes. Notably, MRA treatment resumed in approximately 21.3% of episodes where it had been discontinued during the first 120 days.

## Discussion

In this study we show that patients with hyperkalemia are more often treated with a first- than a second-generation potassium binder. Those treated with a second-generation potassium binder had more comorbidities (e.g., CKD), were more often on dialysis treatment, and had higher persistence with potassium binder treatment than those who had been prescribed a first-generation binder. Patients treated with RAASi had more comorbidities and were more often treated with other medications indicated for management of cardiovascular, metabolic, and renal diseases, compared to those who were not. Both generations of potassium binders reduced serum potassium concentrations within 15 days after the initiation of treatment and sustained normokalemia throughout the remainder of the follow-up period. Although most potassium binder treatment episodes resulted in sustained normokalaemia, RAASi treatment was still discontinued, or the dose reduced, within the 120 days after the initiation of potassium binder treatment in a large proportion of the treatment episodes.

To our best knowledge, only one previous study has assessed treatment with first-and second-generation potassium binders in contemporary routine care, a study of a somewhat smaller population and limited to Stockholm County, Sweden [14]. The findings in this present study largely confirm the findings from that study by Gonzalez-Ortiz et al. [14], which reported that patients using second-generation potassium binders have higher persistence with their potassium binder treatment. There can be several reasons behind higher persistence in patients treated with second-generation potassium binders compared to those with first generation binders. Firstly, the second-generation potassium binders may be more pleasant-tasting enabling adherence [16]. Secondly, occurance of adverse events may differ between the first- and second-generation potassium binders, although evidence supporting this from previous studies is inconclusive [17, 18]. Additionally, differences in baseline covariates between the patient groups may also influence persistence patterns.The findings in this current study also confirm findings from Gonzalez-Ortiz et al. [14] showing that first- and second-generation potassium binders both lowered serum potassium concentrations to a similar extent within the first 15 days after treatment initiation, maintaining normokalemia thereafter. The present study found a similar serum-potassium lowering effect when an as-treated analysis was performed. Covering a longer and more recent time-period, the results from this present study support that the findings from Gonzalez-Ortiz et al. are generalizable to a wider Swedish population, which in turn may be generalizable to other countries that have public healthcare systems similar to that in Sweden.

While this present study didn’t make any formal comparisons of relative effectiveness between different generations of the potassium binders, Gonzalez-Ortiz et al. found no statistical difference between the level that first- and second-generation potassium binders lowered serum potassium concentrations to in routine clinical care [14]. However, they did note that the odds ratios for reaching normokalemia within the first 15 days after the initiation of potassium binder treatment and maintaining it thereafter suggest that a first-generation potassium binder may be more effective [14]. Although Gonzalez-Ortiz et al. didn’t find any statistical differences in the rate of adverse effects between different potassium binder, they did report that the absolute risks were numerically higher in patients treated with a first-generation binder, and that the relative risks were consistently lower in patients treated with a second-generation binder [14].

This present study provides a novel assessment of changes in RAASi inhibitor treatment following the initiation of potassium binder treatment. Given that the potassium binder treatment addressed hyperkalemia, rapidly achieving normokalemia, within the first 15 days after the initiation of most treatment episodes, it may seem a bit premature that RAASi treatment was discontinued, or the dose reduced, in large proportions of treatment episodes during the first 120 days of treatment initiation. However, this trend may simply be a reflection of the traditional response to hyperkalemia in patients using RAASi [3, 19]. Additionally, it may also be a reflection of the common underuse of guideline directed medical therapy in conditions where it is indicated or limitations in access to potassium testing as a follow-up to treatment changes [20].

### Strengths and limitations

This study had access to national health registries detailing most incidences of healthcare and medication use by each registered resident of Sweden. Through an extensive data extraction procedure utilizing the unique personal identification number that is consistently used across all levels of public and private health care, the national health records were linked to relevant clinical data (e.g., serum potassium levels) from the departments of clinical chemistry across six regions in mid-Sweden. To our best knowledge, no other study has linked the Swedish national health records with regional data from such a considerable number of departments of clinical chemistry servicing ~ 18% of Sweden’s residents. Despite its strength in its capacity to identify and characterize patients treated with potassium binders, and to detail changes in potassium binder treatment, serum potassium levels, and RAASi treatment thereafter, several limitations must be acknowledged. Most of the potassium binder treatment episodes followed during the study period were episodes of first-generation potassium binder treatment. While this disparity in treatment during the study period might not limit the characterizations of the patients, it is likely that it does limit the capability to perform comparisons of effectiveness between first- and second-generation potassium binders. Indeed, compared to patients treated with a first-generation potassium binder, there were relatively few available follow-up measures of serum potassium levels after the baseline measure for patients treated with a second-generation potassium binder. Additionally, there were incidences where the prescribed daily dose of a potassium binder, determined by semi-manually analyzing the free-text prescribed dose, couldn’t be interpreted and the mean dose for the specific potassium binder was subsequently imputed. Consequently, the analyses of the effect of potassium binder treatment on potassium levels couldn’t be adjusted for differences in medication dose. Moreover, this limits estimates of persistence with potassium binder treatment. It is also important to emphasise that the potassium values were only available for those in the *Mid-Sweden Cohort* only (although representing ~ 18% of Sweden’s residents), and therefore the conclusion that potassium binder treatment resulted in reduced serum potassium concentrations is based on this cohort only. However, given the similarities in patient characteristics between the *National* and *Mid-Sweden Cohorts*, and supported by a previous smaller study carried out in a different region in Sweden, it is unlikely that potassium binder treatment would elicit different changes in potassium levels between these cohorts [14].

## Conclusion

Treatment with a first and a second-generation potassium binder lowered serum potassium levels to a similar extent within 15 days after the initiation of treatment, but second-generation potassium binders were more often used for younger patients that had more comorbidities and who were slightly more persistent with treatment, when compared to patients prescribed first generation potassium binders. Since normokalemia was achieved rapidly after initiation of most treatment episodes, it raises the possibility that reduction of dose or discontinuation of cardioprotective RAASi treatment around the same period may seem a bit premature.

## Electronic supplementary material

Below is the link to the electronic supplementary material.

Supplementary Material 1

## Data Availability

Requests for data can be made to Maria K Svensson. Data for this research project will be available upon reasonable request after performed legal assessment to ensure that confidentiality of the data will be maintained.
