# Supplementary material for: Potassium binders in clinical practice: understanding potassium binder use in contemporary Swedish healthcare—the DEMONSTRATE database
Source: BMC Nephrol. 2025 Apr 28;26:213. doi: 10.1186/s12882-025-04146-8 (PMC12036272; doi:10.1186/s12882-025-04146-8)
Supplement: Supplementary file 1 — Supplementary Material 1 [file 12882_2025_4146_MOESM1_ESM.docx]

Supplemental Material

Contents

[Supplemental Methods 1 2](#_Toc182822180)

[Supplemental Methods 2 3](#_Toc182822181)

[Supplemental Methods 3 4](#_Toc182822182)

[Supplemental Methods 4 5](#_Toc182822183)

[Supplemental Methods 5 6](#_Toc182822184)

[Table S1. 8](#_Toc182822185)

[Table S2. 10](#_Toc182822186)

[Table S3. 12](#_Toc182822187)

[Supplemental Figure 1. 14](#_Toc182822188)

Supplemental Methods 1

Record of treatment with sodium polystyrene sulfonate, patiromer, and sodium zirconium cyclosilicate was searched for in the Swedish Prescribed Drug Register using the ATC codes, V03AE01, V03AE09, and V03AE10, respectively.

Supplemental Methods 2

For any given patient, the first dispensation of a potassium binder during the study period initiated their first potassium binder treatment episode. If there was a subsequent dispensation of a potassium binder for the given patient within ≤120 days of the initial dispensation, then the treatment episode was extended by another 120 days. Thus, one treatment episode could include multiple dispensations. A period of 120 days was chosen as the Swedish reimbursement system allows for a medication supply covering three months of treatment to be dispensed with a single prescription. A one-month grace period was then added to account for variation in medication adherence (e.g., due to missed days of treatment and subsequent surplus medication supply). If there wasn’t an additional dispensation within ≤120 days of the previous dispensation, then the treatment episode ended. If a subsequent dispensation was made >120 days following the previous dispensation, then that dispensation initiated a new treatment episode, which could also be extended by additional dispensations according to the same rules detailed above.

# Supplemental Methods 3

Listed below are the ICD-10 codes and clinical procedure codes used to identify prevalent comorbidities of interest in each patient. The codes listed below were used to identify prevalent conditions for each patient. Patients were classified as having a condition if a corresponding record was found at any time prior to or on the index date.

| **Diagnosis or Procedure** | **ICD-10 codes** | **Procedure codes** |
| --- | --- | --- |
| Heart failure | I50, I11.0, I13.0, I13.2, I25.5, I42.0, I42.6, I42.7, I42.9, I43.1 |  |
| Chronic kidney disease | E10.2, E11.2, E12.2, E13.2, E14.2, N08.3, N17.X, N18.X, N19.X, I12.0, I12.9, I13.1, I13.2, I13.9, Z49.1, Z49.2, Z99.2 | JAK10, TJA33, TJA35, DJ008, DR013, DR014, DR015, DR016, DR017, DR023, DR024, DR055, DR056, DR060, DR061, KAS10, KAS20, QF006 |
| Diabetes mellitus | E10, E11, E12, E13, E14 |  |
| Hypertension | I10, I11, I12, I13, I15 |  |
| Dialysis | Z49.1, Z49.2, Z99.2 | DR013, DR014, DR015, DR016, DR017, DR023, DR024, DR055, DR056, DR060, DR061 |

# Supplemental Methods 4

Listed below are the ATC codes used to identify medication use of interest by each patient during the 120 days prior to and on the index date.

| **Medication** | **ATC codes** |
| --- | --- |
| RASi (ACEi/ARB/ARNi) | C09 |
| RAASi (ACEi, ARB, ARNi, MRA) | C09, C03DA |
| Antihypertensives | C02, C09, C03A, C07, C08C |
| Diuretics | C03A, C03B, C03C, C03E |
| SGLT2i | A10BK, A10DB15, A10DB16, A10DB19, A10DB20, A10DB21, A10DB23, A10DB24 |

RASi denotes renin-angiotensin system inhibitors; RAASi, renin-angiotensin aldosterone system inhibitors; ACEi, angiotensin-converting enzyme inhibitors; ARB, angiotensin receptor blockers; ARNi, angiotensin receptor-neprilysin inhibitors; MRA, mineralocorticoid receptor antagonists; SGLT2i, sodium-glucose cotransporter 2 inhibitors.

# Supplemental Methods 5

Changes in renin-angiotensin-aldosterone system (RAAS) inhibitor use were analyzed separately for angiotensin-converting enzyme (ACE) inhibitors/angiotensin receptor blockers (ARB) and mineralocorticoid receptor antagonists (MRA). These analyses were restricted to patients with at least 120 days of follow-up and to potassium binder episodes where ACE inhibitors/ARB or MRA were used at the time of potassium binder initiation (index date). Treatment status at the index date was determined based on the most recent pharmacy dispensation of ACE inhibitors/ARB and MRA prior to the index date.

For each episode, changes in ACE inhibitor/ARB and MRA treatment were assessed at 120- and 240-days post-index. If a pharmacy dispensation provided medication coverage up to day 120, the episode was classified as still being treated with ACE inhibitors/ARB or MRA. If coverage did not extend to day 120, the episode was categorized as discontinued. The same approach was applied for day 240 post-index. To evaluate changes in dose, all prescribed doses were converted to a proportion of the target dose for each substance. For example, a prescribed dose of 50 mg of Losartan was considered 33.3% of the target dose, as the target dose for Losartan is 150 mg.

The following changes were assessed:

1. Maintained ACE inhibitor/ARB or MRA: Defined as no observed change in the proportion of the target dose.
2. Decreased ACE inhibitor/ARB or MRA: Defined as a reduction in the proportion of the target dose.
3. Increased ACE inhibitor/ARB or MRA: Defined as an increase in the proportion of the target dose.
4. Discontinued ACE inhibitor/ARB or MRA: Defined as no pharmacy dispensation observed between index and 120 days, or between 120 days and 240 days.

The following target doses have been used:

| **ATC code** | **Substance name** | **Target dose** |
| --- | --- | --- |
| C09AA02 | Enalapril | 20 mg |
| C09CA01 | Losartan | 150 mg |
| C03DA01 | Spironolactone | 50 mg |
| C09AA05 | Ramipril | 10 mg |
| C09CA04 | Irbesartan | 300 mg |
| C09DX04 | Valsartan and Sacubitril | 320 mg |
| C03DA04 | Eplerenone | 50 mg |
| C09DA06 | Candesartan and diuretics | 32 mg |
| C09BA02 | Enalapril and diuretics | 20 mg |
| C09DA01 | Losartan and diuretics | 150 mg |
| C09CA03 | Valsartan | 320 mg |
| C09AA03 | Lisinopril | 20 mg |
| C09CA07 | Telmisartan | 80 mg |
| C09DA04 | Irbesartan and diuretics | 300 mg |
| C09DA03 | Valsartan and diuretics | 320 mg |
| C09AA01 | Captopril | 150 mg |
| C09BA05 | Ramipril and diuretics | 10 mg |
| C09DA07 | Telmisartan and diuretics | 80 mg |
| C09DB01 | Valsartan and amlodipine | 320 mg |
| C09BA03 | Lisinopril and diuretics | 20 mg |
| C09CA02 | Eprosartan | 600 mg |
| C09BA08 | Cilazapril and diuretics | 5 mg |
| C09AA09 | Fosinopril | 20 mg |
| C09BA06 | Quinapril and diuretics | 20 mg |
| C09AA08 | Cilazapril | 2.5 mg |
| C09AA06 | Quinapril | 20 mg |
| C09AA10 | Trandolapril | 2 mg |
| C09DA02 | Eprosartan and diuretics | 600 mg |
| C03DA05 | Finerenone | 20 mg |

Table S1. Characteristics of the patients included in the *National* and *Mid-Sweden Cohorts* stratified by those who used first- or second-generation potassium binders at the beginning of each potassium binder treatment episode

|  |  | National cohort | National cohort | Mid-Sweden cohort | Mid-Sweden cohort |
| --- | --- | --- | --- | --- | --- |
|  |  | First generation | Second generation | First generation | Second generation |
| **N, potassium binder treatment episodes** |  | 22,069 | 1,823 | 4,633 | 227 |
| **N, patients** |  | 13,428 | 1,275 | 3,095 | 155 |
| **Age, years, mean (SD)** | Mean (SD) | 70.6 (14.8) | 65.2 (16.5) | 71.8 (14.5) | 66.7 (14.1) |
|  | Median (IQR) | 73.0 (63.0-81.0) | 69.0 (55.0-78.0) | 75.0 (64.0-82.0) | 70.0 (58.0-77.0) |
| **Women, N (%)** |  | 7,278 (33.0%) | 635 (34.8%) | 1,573 (34.0%) | 80 (35.2%) |
| **Laboratory measurements, mean (SD)** |  |  |  |  |  |
| Baseline potassium level (mmol/L) |  | N/A | N/A | 5.6 (0.7) | 5.6 (0.7) |
| eGFR, ml/min/1.73m^2^ | Mean (SD) | N/A | N/A | 23.8 (17.1) | 18.9 (15.6) |
|  | Median (IQR) | N/A | N/A | 19.2 (10.4-31.5) | 13.0 (7.8-25.9) |
| eGFR, ml/min/1.73m^2^ (excluding patients with dialysis) | Mean (SD) | N/A | N/A | 27.9 (17.1) | 26.5 (16.7) |
|  | Median (IQR) | N/A | N/A | 22.9 (15.7-36.2) | 22.2 (15.3-34.2) |
| **Comorbidities, N (%)** |  |  |  |  |  |
| Heart failure |  | 7,596 (34.4%) | 729 (40.0%) | 1,782 (38.5%) | 94 (41.4%) |
| Hypertension |  | 19,185 (86.9%) | 1,609 (88.3%) | 4,097 (88.4%) | 201 (88.5%) |
| Diabetes mellitus |  | 10,014 (45.4%) | 853 (46.8%) | 2,164 (46.7%) | 108 (47.6%) |
| Type 1 diabetes mellitus |  | 4,556 (20.6%) | 450 (24.7%) | 1,008 (21.8%) | 73 (32.2%) |
| Type 2 diabetes mellitus |  | 9,511 (43.1%) | 795 (43.6%) | 2,074 (44.8%) | 105 (46.3%) |
| Chronic kidney disease (based on diagnosis and procedure codes) |  | 18,370 (83.2%) | 1,606 (88.1%) | 3,745 (80.8%) | 205 (90.3%) |
| - eGFR < 60 ml/min |  | N/A | N/A | 4,409 (95.2%) | 223 (98.2%) |
| - eGFR < 15 ml/min |  | N/A | N/A | 1,781 (38.4%) | 120 (52.9%) |
| **Dialysis, N (%)** |  |  |  |  |  |
| Hemodialysis |  | 5,648 (25.6%) | 647 (35.5%) | 1,069 (23.1%) | 86 (37.9%) |
| Peritoneal dialysis |  | 831 (3.8%) | 83 (4.6%) | 156 (3.4%) | 9 (4.0%) |
| **Medication use, N (%)** |  |  |  |  |  |
| RASi (ACEi, ARB) |  | 13,589 (61.6%) | 1,247 (68.4%) | 3,051 (65.9%) | 146 (64.3%) |
| RAASi (ACEi, ARB, MRA) |  | 14,105 (63.9%) | 1,291 (70.8%) | 3,220 (69.5%) | 148 (65.2%) |
| ARNi |  | 357 (1.6%) | 128 (7.0%) | 119 (2.6%) | 10 (4.4%) |
| MRA |  | 2,655 (12.0%) | 341 (18.7%) | 855 (18.5%) | 33 (14.5%) |
| Antihypertensive medications |  | 19,658 (89.1%) | 1,677 (92.0%) | 4,228 (91.3%) | 207 (91.2%) |
| Diuretics |  | 10,905 (49.4%) | 865 (47.4%) | 2,307 (49.8%) | 93 (41.0%) |
| Beta Blockers |  | 14,267 (64.6%) | 1,282 (70.3%) | 3,182 (68.7%) | 168 (74.0%) |
| SGLT2i |  | 528 (2.4%) | 138 (7.6%) | 122 (2.6%) | 6 (2.6%) |

eGFR denotes estimated glomerular filtration rate; RASi, renin-angiotensin system inhibitors; ACEi, angiotensin-converting enzyme inhibitors; ARB, angiotensin receptor blockers; RAASi, renin-angiotensin aldosterone system inhibitors; ARNi, angiotensin receptor-neprilysin inhibitors; MRA, mineralocorticoid receptor antagonists; SGLT2i, sodium-glucose cotransporter-2 inhibitors.

Table S2. Characteristics of the patients included in the *National* and *Mid-Sweden Cohorts* stratified by those who used or didn’t use renin-angiotensin aldosterone system inhibitors at the beginning of each potassium binder treatment episode

|  |  | National cohort | National cohort | Mid-Sweden cohort | Mid-Sweden cohort |
| --- | --- | --- | --- | --- | --- |
|  |  | Used RAASi | **Didn’t use RAASi** | Used RAASi | Didn’t use RAASi |
| **N, potassium binder treatment episodes** |  | 15,396 | 8,496 | 3,368 | 1,492 |
| **N, patients** |  | 9,911 | 5,698 | 2,358 | 1,058 |
| **Age, years, mean (SD)** | Mean (SD) | 70.0 (14.9) | 70.6 (15.1) | 71.8 (14.4) | 71.2 (14.9) |
|  | Median (IQR) | 73.0 (62.0-81.0) | 73.0 (62.0-81.0) | 75.0 (64.0-82.0) | 74.0 (63.0-82.0) |
| **Women, N (%)** |  | 4,981 (32.4%) | 2,932 (34.5%) | 1,116 (33.1%) | 537 (36.0%) |
| **Laboratory measurements, mean (SD)** |  |  |  |  |  |
| Baseline potassium level (mmol/L) |  | N/A | N/A | 5.6 (0.7) | 5.6 (0.8) |
| eGFR, ml/min/1.73m^2^ | Mean (SD) | N/A | N/A | 25.3 (16.7) | 19.6 (17.3) |
|  | Median (IQR) | N/A | N/A | 21.2 (12.5-33.9) | 13.1 (8.3-24.0) |
| eGFR, ml/min/1.73m^2^ (excluding patients with dialysis) | Mean (SD) | N/A | N/A | 28.8 (16.2) | 24.9 (19.1) |
|  | Median (IQR) | N/A | N/A | 24.2 (17.4-37.4) | 18.5 (11.8-30.8) |
| **Comorbidities, N (%)** |  |  |  |  |  |
| Heart failure |  | 5,979 (38.8%) | 2,346 (27.6%) | 1,445 (42.9%) | 431 (28.9%) |
| Hypertension |  | 13,913 (90.4%) | 6,881 (81.0%) | 3,084 (91.6%) | 1,214 (81.4%) |
| Diabetes mellitus |  | 7,557 (49.1%) | 3,310 (39.0%) | 1,722 (51.1%) | 550 (36.9%) |
| Type 1 diabetes mellitus |  | 3,429 (22.3%) | 1,577 (18.6%) | 811 (24.1%) | 270 (18.1%) |
| Type 2 diabetes mellitus |  | 7,172 (46.6%) | 3,134 (36.9%) | 1,658 (49.2%) | 521 (34.9%) |
| Chronic kidney disease (based on diagnosis and procedure codes) |  | 12,510 (81.3%) | 7,466 (87.9%) | 2,640 (78.4%) | 1,310 (87.8%) |
| - eGFR < 60 ml/min |  | 4,162 (92.0%) | 1,902 (91.5%) | 3,209 (95.3%) | 1,423 (95.4%) |
| - eGFR < 15 ml/min |  | 1,221 (27.0%) | 977 (47.0%) | 1,069 (31.7%) | 832 (55.8%) |
| **Dialysis, N (%)** |  |  |  |  |  |
| Hemodialysis |  | 3,110 (20.2%) | 3,185 (37.5%) | 633 (18.8%) | 522 (35.0%) |
| Peritoneal dialysis |  | 579 (3.8%) | 335 (3.9%) | 107 (3.2%) | 58 (3.9%) |
| **Medication use, N (%)** |  |  |  |  |  |
| RASi (ACEi, ARB) |  | 14,836 (96.4%) | 0 (0.0%) | 3,197 (94.9%) | 0 (0.0%) |
| RAASi (ACEi, ARB, MRA) |  | 15,396 (100.0%) | 0 (0.0%) | 3,368 (100.0%) | 0 (0.0%) |
| ARNi |  | 485 (3.2%) | 0 (0.0%) | 129 (3.8%) | 0 (0.0%) |
| MRA |  | 2,996 (19.5%) | 0 (0.0%) | 888 (26.4%) | 0 (0.0%) |
| Antihypertensive medications |  | 15,307 (99.4%) | 6,028 (71.0%) | 3,350 (99.5%) | 1,085 (72.7%) |
| Diuretics |  | 8,454 (54.9%) | 3,316 (39.0%) | 1,867 (55.4%) | 533 (35.7%) |
| MRA |  | 2,996 (19.5%) | 0 (0.0%) | 888 (26.4%) | 0 (0.0%) |
| Beta Blockers |  | 10,810 (70.2%) | 4,739 (55.8%) | 2,480 (73.6%) | 870 (58.3%) |
| SGLT2i |  | 615 (4.0%) | 51 (0.6%) | 122 (3.6%) | 6 (0.4%) |

eGFR denotes estimated glomerular filtration rate; RASi, renin-angiotensin system inhibitors; ACEi, angiotensin-converting enzyme inhibitors; ARB, angiotensin receptor blockers; ARNi, angiotensin receptor-neprilysin inhibitors; MRA, mineralocorticoid receptor antagonists; SGLT2i, sodium-glucose cotransporter-2 inhibitors.

Table S3. Characteristics of the patients included in the *National* and *Mid-Sweden Cohorts* stratified by those who used or didn’t use mineralocorticoid receptor antagonists at the beginning of each potassium binder treatment episode

|  |  | National cohort | National cohort | Mid-Sweden cohort | Mid-Sweden cohort |
| --- | --- | --- | --- | --- | --- |
|  |  | Used MRA | Didn’t use MRA | Used MRA | Didn’t use MRA |
| **N, potassium binder treatment episodes** |  | 2,996 | 20,896 | 888 | 3,972 |
| **N, patients** |  | 2,405 | 12,221 | 708 | 2,564 |
| **Age, years, mean (SD)** | Mean (SD) | 74.0 (12.4) | 69.7 (15.2) | 74.9 (12.3) | 70.9 (14.9) |
|  | Median (IQR) | 76.0 (68.0-83.0) | 73.0 (61.0-80.0) | 77.0 (70.0-83.0) | 74.0 (62.0-82.0) |
| **Women, N (%)** |  | 1,127 (37.6%) | 6,786 (32.5%) | 324 (36.5%) | 1,329 (33.5%) |
| **Laboratory measurements, mean (SD)** |  |  |  |  |  |
| Baseline potassium level (mmol/L) |  | N/A | N/A | 5.7 (0.7) | 5.6 (0.7) |
| eGFR, ml/min/1.73m^2^ | Mean (SD) | N/A | N/A | 30.0 (17.1) | 22.1 (16.8) |
|  | Median (IQR) | N/A | N/A | 26.3 (18.0-41.0) | 17.3 (9.5-28.6) |
| eGFR, ml/min/1.73m^2^ (excluding patients with dialysis) | Mean (SD) | N/A | N/A | 33.1 (16.2) | 26.4 (17.0) |
|  | Median (IQR) | N/A | N/A | 29.2 (20.8-43.3) | 21.4 (14.4-33.3) |
| **Comorbidities, N (%)** |  |  |  |  |  |
| Heart failure |  | 2,050 (68.4%) | 6,275 (30.0%) | 622 (70.0%) | 1,254 (31.6%) |
| Hypertension |  | 2,634 (87.9%) | 18,160 (86.9%) | 794 (89.4%) | 3,504 (88.2%) |
| Diabetes mellitus |  | 1,518 (50.7%) | 9,349 (44.7%) | 448 (50.5%) | 1,824 (45.9%) |
| Type 1 diabetes mellitus |  | 515 (17.2%) | 4,491 (21.5%) | 155 (17.5%) | 926 (23.3%) |
| Type 2 diabetes mellitus |  | 1,474 (49.2%) | 8,832 (42.3%) | 442 (49.8%) | 1,737 (43.7%) |
| Chronic kidney disease (based on diagnosis and procedure codes) |  | 1,919 (64.1%) | 18,057 (86.4%) | 573 (64.5%) | 3,377 (85.0%) |
| eGFR < 60 ml/min |  | 976 (88.8%) | 5,088 (92.5%) | 830 (93.5%) | 3,802 (95.7%) |
| eGFR < 15 ml/min |  | 187 (17.0%) | 2,011 (36.6%) | 175 (19.7%) | 1,726 (43.5%) |
| **Dialysis, N (%)** |  |  |  |  |  |
| Hemodialysis |  | 348 (11.6%) | 5,947 (28.5%) | 113 (12.7%) | 1,042 (26.2%) |
| Peritoneal dialysis |  | 96 (3.2%) | 818 (3.9%) | 22 (2.5%) | 143 (3.6%) |
| **Medication use, N (%)** |  |  |  |  |  |
| RASi (ACEi, ARB) |  | 2,436 (81.3%) | 12,400 (59.3%) | 717 (80.7%) | 2,480 (62.4%) |
| RAASi (ACEi, ARB, MRA) |  | 2,996 (100.0%) | 12,400 (59.3%) | 888 (100.0%) | 2,480 (62.4%) |
| ARNi |  | 286 (9.5%) | 199 (1.0%) | 86 (9.7%) | 43 (1.1%) |
| MRA |  | 2,996 (100.0%) | 0 (0.0%) | 888 (100.0%) | 0 (0.0%) |
| Antihypertensive medications |  | 2,907 (97.0%) | 18,428 (88.2%) | 870 (98.0%) | 3,565 (89.8%) |
| Diuretics |  | 2,046 (68.3%) | 9,724 (46.5%) | 609 (68.6%) | 1,791 (45.1%) |
| Beta Blockers |  | 2,522 (84.2%) | 13,027 (62.3%) | 778 (87.6%) | 2,572 (64.8%) |
| SGLT2i |  | 279 (9.3%) | 387 (1.9%) | 62 (7.0%) | 66 (1.7%) |

eGFR denotes estimated glomerular filtration rate; RASi, renin-angiotensin system inhibitors; ACEi, angiotensin-converting enzyme inhibitors; ARB, angiotensin receptor blockers; ARNi, angiotensin receptor-neprilysin inhibitors; MRA, mineralocorticoid receptor antagonists; SGLT2i, sodium-glucose cotransporter-2 inhibitors.

Supplemental Figure 1. Potassium levels after initiating potassium binder treatment (as treated analysis). In this sensitivity analysis, we conducted an as-treated analysis that included only potassium values measured while patients had an active supply of potassium binder treatment at the time of their potassium measurement. The scatter plot shows individual potassium measurements over time in patients treated with first-generation or second-generation binders. The solid lines represent predicted potassium levels derived from a mixed-effects model adjusted for age, sex, heart failure, hypertension, chronic kidney disease, diabetes, use of RAASi and MRA, and baseline potassium levels. Prediction mean with error bars reflecting 95% confidence intervals are presented for the median patient at index and 15-, 30-, 45-, and 60-days post-index).
